# Catechol-o-methyltransferase inhibitor tolcapone improves learning and memory in naïve but not in haloperidol challenged rats

**DOI:** 10.22038/ijbms.2019.33025.7890

**Published:** 2019-06

**Authors:** Anita Mihaylova, Hristina Zlatanova, Nina Doncheva, Delian Delev, Ilia Kostadinov

**Affiliations:** 1 Department of Pharmacology and Drug Toxicology, Faculty of Pharmacy, Medical University Plovdiv, 15A Vassil Aprilov Blvd., Plovdiv 4002, Bulgaria; 2 Department of Pharmacology and Clinical Pharmacology, Faculty of Medicine, Medical University Plovdiv, 15A Vassil Aprilov Blvd., Plovdiv 4002, Bulgaria

**Keywords:** COMT, Dopamine, Hippocampus, Prefrontal cortex, Spatial memory, Tolcapone, Working memory

## Abstract

**Objective(s):**

Dopamine plays an important role in cognitive functions. Inhibition of the dopamine-degrading enzyme catechol-O-methyltransferase (COMT) may have beneficial effects. Our aim was to assess the effect of COMT inhibitor tolcapone (TCP) on learning and memory in naïve and haloperidol-challenged rats.

**Materials and Methods:**

Male Wistar rats were divided into 9 groups (n=8): naïve-saline, tolcapone 5; 15 and 30 mg/kg BW; haloperidol (HP) challenged-saline, haloperidol, haloperidol+tolcapone 5; 15 and 30 mg/kg BW. Two-way active avoidance test (TWAA), elevated T-maze, and activity cage were performed. Observed parameters were: number of conditioned responses (CR) and unconditioned responses (UCR), working memory index, and vertical and horizontal movements.

**Results:**

Naïve rats with 30 mg/kg BW TCP had a significantly increased number of CR and UCR during the long-term memory test. The animals with 5 mg/kg BW TCP significantly increased the number of UCR during the two retention tests. In haloperidol-challenged rats, the three experimental groups decreased the number of CR and UCR during the learning session and the two memory tests, compared to the saline group. There was no significant difference between the HP-challenged rats treated with TCP and the haloperidol control group. All experimental naïve groups had significantly increased working memory index whereas none of the HP-challenged groups showed significant increase in this parameter.

**Conclusion:**

Our results demonstrate that in naïve rats tolcapone improves memory in the hippocampal-dependent TWAA task and spatial working memory in T-maze.

## Introduction

Parkinson’s disease (PD) is the second most common neurodegenerative disorder after Alzheimer’s disease (1, 2). The major pathogenic feature of the disease is progressive loss of 50-70% of dopamine (DA) neurons in substantia nigra pars compacta (SNpc), (3). PD is usually characterized by its motor symptoms (MS): rigidity, postural tremor, and bradykinesia (4). The non-motor symptoms (NMS), including cognitive deficits, sleep disturbances, and autonomic and sensory dysfunction are increasingly recognized in the last few decades (3). 

The cognitive impairment varies from mild cognitive decline to PD-related dementia (5), and its pathogenesis remains unknown (6). Some clinical studies suggest that the cognitive decline might be explained by alterations in the dopaminergic mediation in the prefrontal cortex, hippocampus, and amygdala (7). Dopamine plays an important role in a variety of hippocampal functions, such as cognition, learning, and memory processes (8). In the last two decades, researchers have found hippocampal atrophy in PD patients with memory decline (9). These clinical results are supported by preclinical data that show that impaired behavioral and cognitive tasks in experimental animals with PD are associated with changes in the hippocampus and prefrontal cortex (6). 

The COMT enzyme catalyzes extraneuronal metabolism of the catecholamines one of which is dopamine. The action of dopamine in the synaptic space is terminated either through active uptake by dopamine transporters, diffusion out of the synaptic cleft or metabolism by COMT (10). In the prefrontal cortex, COMT plays a more important role in dopamine metabolism due to the low levels of dopamine transporters. However, in the striatum, the uptake by dopamine transporter is the primary way of terminating dopamine action (11). COMT genotype in humans is associated with cognitive functions. The most widely studied variation is the valine-to-methionine substitution at codon 158. COMT Val^158^ has a higher enzyme activity with decreased cognitive stability (12). 

Second generation COMT inhibitors, such as Tolcapone, are considered adjuncts to the standard treatment of PD. Clinical and preclinical data show that tolcapone improves memory functions. Administration of COMT inhibitors has a strong impact on executive functions and cognition in healthy humans and patients with PD. (13, 14). The beneficial effects of low COMT activity on cognitive performance are associated with its effects on the prefrontal cortex (15). Nevertheless, the COMT effect in other brain areas remains unexplored. In this regard, the hippocampus is a good candidate region due to its involvement in learning and memory processes (16).

The COMT enzyme activity in rats is close to the human COMT Val^158^ form (15), making them appropriate objects for exploring the effect of COMT inhibition on memory functions. Recent research suggests that the hippocampal function may facilitate two-way active avoidance conditioning, therefore the active avoidance test could be used to assess learning and memory processes that are associated with the hippocampal functioning (17). Haloperidol is an antagonist of D_2_/D_3_ receptors. Currently, there is no information about the effect of tolcapone on memory in the TWAA task and the impact of haloperidol challenge on this effect. 

The aim of our study was to evaluate the effect of tolcapone on memory and learning using two behavioral tests - TWAA task which is hippocampal dependent and T-maze task, where behavioral responses are associated with neuronal activity in the prefrontal cortex. The second aim was to assess the possible role of D2-like receptors in the observed effects through haloperidol blockage of D_2_/D_3_ receptors.

## Materials and Methods

***Ethical statement***

 The following experimental procedures were carried out in accordance with the European Convention for Protection of Vertebrate Animals used for experimental and other scientific purposes. For this study we obtained permission from the Ethics Committee at the Medical University of Plovdiv (protocol No. 2/19.04.2018) and Animal Health and Welfare Directorate of the Bulgarian Food Safety Agency (permit No. 4/09.12.2015).

***Drugs***

Tolcapone (TCP) (3,4-Dihydroxy-4′-methyl-5-nitrobenzophenone) was purchased from Sigma-Aldrich and haloperidol (HP) from Sopharma (Bulgaria).

***Animals***

Adult male Wistar rats (200±20 g body weight) were used in this study. They were housed in groups of 8 per cage under standard laboratory conditions (12 hr light-dark cycle, light: 08:00-20:00, temperature 22±2 ^°^C, humidity 55±5%, free access to food and water). 

***Experimental design***

To evaluate the effect of TCP on learning and memory in naïve rats, the animals were divided randomly into 4 groups (n=8) as follows: 

Group 1: control group: saline 0.1 ml/100 g BW

Group 2: tolcapone 5 mg/kg BW

Group 3: tolcapone 15 mg/kg BW

group 4: tolcapone 30 mg/kg BW

Tolcapone was suspended in saline with a few drops of Tween 80 and administered orally.

To evaluate the effect of TCP on learning and memory in HP-challenged rats, the animals were randomly divided into 5 groups (n=8) as follows: 

Group 1: (control) saline 0,1 ml/100 g BW

Group 2: (negative control) haloperidol 1 mg/kg BW

Group 3: haloperidol+tolcapone 5 mg/kg BW

Group 4: haloperidol+tolcapone 15 mg/kg BW

Group 5: haloperidol+tolcapone 30 mg/kg BW

All animals were pretreated with TCP for 7 days. HP was administered intraperitoneally (IP) only during the testing days 60 min before the tests. At this dose it causes dopamine striatal depletion manifested within 1 hr of the injection (18). TCP was administered 60 min before HP.

**Figure 1:**
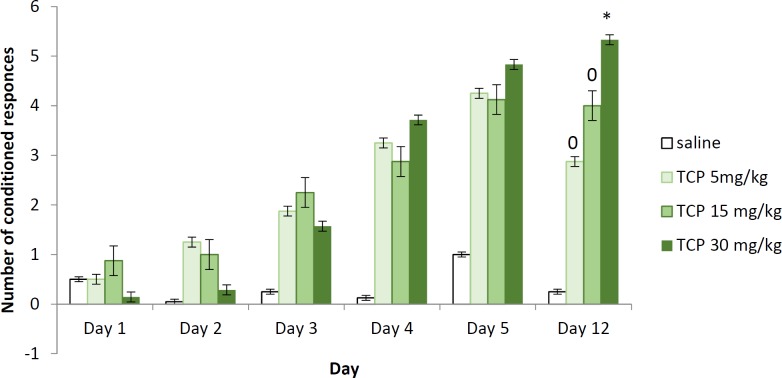
Two-way active avoidance test in naïve rats treated with tolcapone 5, 15, and 30 mg/kg BW (conditioned responses). Data are expressed as mean±SEM (n=8). ANOVA test for comparisons between groups

*P<0.05 compared to saline; ^0^ *P<*0.05 when compared to TCP 30 mg/kg BW; TCP=tolcapone

**Figure 2:**
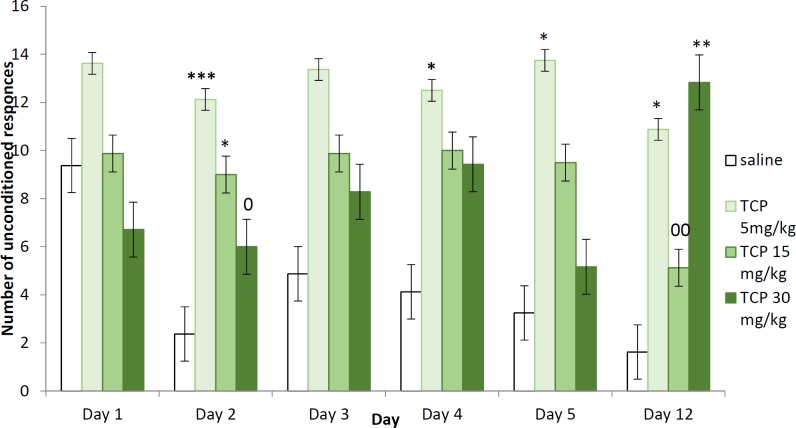
Two-way active avoidance test in naïve rats treated with tolcapone 5, 15, and 30 mg/kg BW (unconditioned responses). Data are expressed as means±SEM (n=8). ANOVA test for comparisons between groups

*P<0.05 compared to saline; **P<0.01 compared to saline; ***P<0.001 compared to saline; ^0 ^*P<*0.05 compared to TCP 5 mg/kg BW; ^00^ *P<*0.05 compared to TCP 30 mg/kg BW; TCP=tolcapone

**Figure 3:**
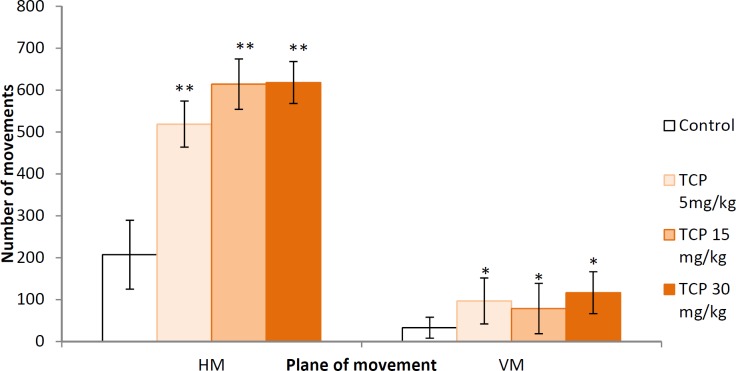
Effects of tolcapone on locomotor activity in naïve rats. Data are expressed as mean±SEM (n=8). ANOVA test for comparisons between groups

*P<0.05 compared to saline; **P<0.001 compared to saline; TCP=tolcapone; HM=horizontal movements; VM=vertical movements

**Figure 4:**
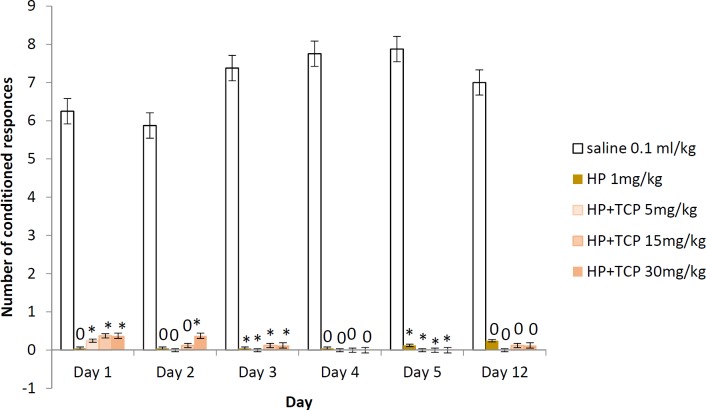
Two-way active avoidance test in haloperidol-challenged rats treated with tolcapone 5, 15, and 30 mg/kg BW (conditioned responses). Data are expressed as mean±SEM (n=8). ANOVA test for comparisons between groups

*P<0.001 compared to saline; ^0 ^*P<*0.0001 compared to saline; TCP=tolcapone

**Figure 5:**
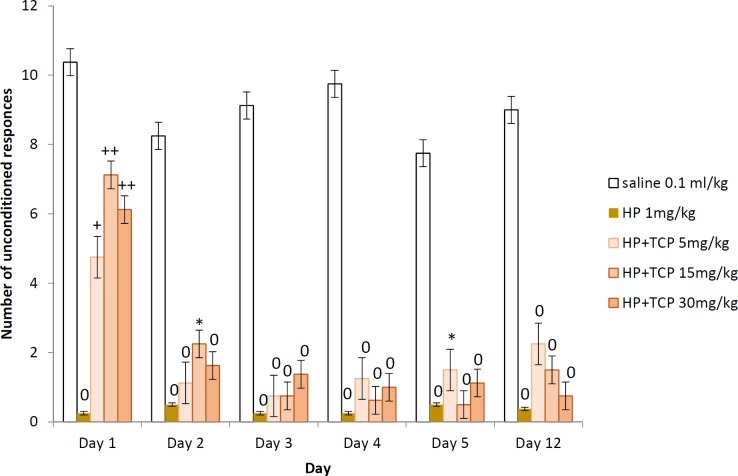
Two-way active avoidance test in haloperidol-challenged rats treated with tolcapone 5; 15 and 30 mg/kg BW (unconditioned responses). Data are expressed as mean±SEM (n=8). ANOVA test for comparisons between groups

*P<0.001 compared to saline; ^0^*P<*0.0001 compared to saline; ^+^*P<*0.05 compared to saline; ^++^*P<*0.05 compared to haloperidol; TCP=tolcapon5

**Figure 6:**
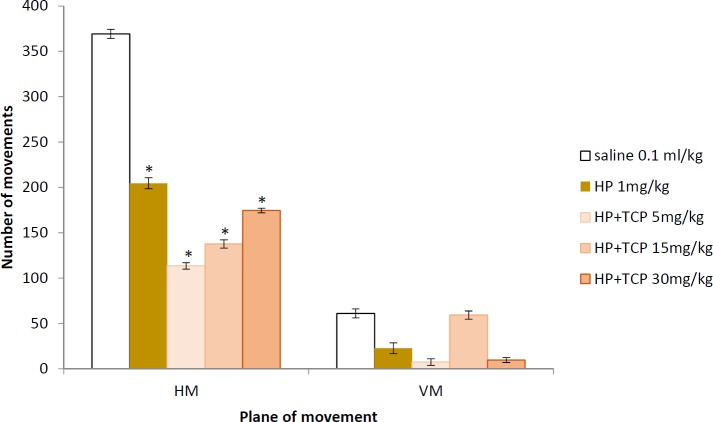
Effects of tolcapone on locomotor activity in naïve rats. Data are expressed as mean±SEM (n=8). ANOVA test for comparisons between groups

*P<0.05 compared to saline; HP=haloperidol; TCP=tolcapone; HM=horizontal movements; VM=vertical movements

**Table 1:** Elevated T-maze test in naïve rats treated with tolcapone 5, 15, and 30 mg/kg bw

Groups	Working memory index
**saline**	0,250±0,185
**TCP 5 mg/kg bw**	0,712±0,275 0
**TCP 15 mg/kg bw**	0,687±0,146 *
**TCP 30 mg/kg bw**	0,800±0,126 **

Data are expressed as mean±SEM (n=8). ANOVA test for comparisons between groups;

TCP=tolcapone.

0 *P*<0.01 compared to saline;

* *P*<0.001 compared to saline;

** *P*<0.0001 compared to saline

**Table 2:** Elevated T-maze test in haloperidol-challenged rats treated with tolcapone 5, 15, and 30 mg/kg bw

Group	Working memory index	Significance
**saline**	0,662±0,159	-
**Haloperidol 1 mg/kg bw**	0,762±0,130	NS vs saline
**HP+TCP 5 mg/kg bw**	0,685±0,121	NS vs saline NS vs haloperidol
**HP** **+** **TCP** ** 1** **5** **mg/kg** **bw**	0,625±0,138	NS vs saline NS vs haloperidol
**HP** **+** **TCP** ** 30 ** **mg** **/** **kg** **bw**	0,637±0,206	NS vs saline NS vs haloperidol

Data are expressed as mean ±SEM (n=8). ANOVA test for comparisons between groups;

TCP=tolcapone; HP=haloperidol; NS=no significance.

***Behavioral tests***

*Two-way active (shuttle) avoidance test*

A fully automated shuttle-box apparatus (Ugo Basile, Comerio-Varese, Italy) was used. The training session involved 30 trials daily for 4 consecutive days. Memory retention tests for short-term and long-term memory were performed on the 5^th^ and 12^th^ days, respectively. Rats were conditioned by using light and buzzer (670 Hz, 70 dB, 6 sec) as conditioned stimuli (CS) and electrical foot shock (0,4 mA, 3 sec) as an unconditioned stimulus (UCS). The interval between the CS and UCS was 12 sec. The following parameters were recorded: number of avoidances (conditioned responses-CR), number of escapes (unconditioned responses-UCR).

*Elevated T-maze (spatial working memory) test*

We used a self-made T-maze to assess spatial working memory in rats. It was 50 cm above ground and had a capital T-shape design with a stem length of 50 cm and an arm length of 40 cm. The test relies on either spontaneous or rewarded alternation. In our study, we used the latter. The animals had restricted food intake 24 hr before the experiment. Each learning session consisted of 11 trials an initial forced trial followed by 10 choice trials. During the forced trial one of the arms was closed and reward pallets were placed in the opposite arm, therefore the animal was forced to enter the baited arm. During choice trials, both arms were opened and accessible, and the reward was available at the same arm as visited in the 1^st^ trial. In the choice trials, the rats had to avoid the unexplored arm, which is their natural instinct, and enter the well-known arm with reward pallets. The animal was placed at the base of the T-shape and arm entries were recorded, when the whole rat was in the arm. There was an inter-trial interval of 5 min. A working memory index was calculated number of correct choices out of the total number of trials.

*Locomotor activity (activity cage) test*

An automatic apparatus (47420 multiple activity cage, Ugo Basile, Italy) was used to assess horizontal and vertical spontaneous movements of the animals. The set-up comprised an electronic unit and an infra-red beam cage complete with two sets of sensor arrays for horizontal and vertical activity. The animal is placed into the plastic cage for 5 min. The movement it makes inside the cage interrupts one or more infra-red beam(s). The beam interruptions are counted and recorded by the electronic device. This test was performed to avoid false positive or false negative results because of increased or decreased locomotor activity, respectively. This is due to the fact that dopamine increases motor activity.

***Statistics***

Statistical analysis was performed by using IBM SPSS Statistics 19.0. All data are expressed as mean±SEM (standard errors of the mean). Data were analyzed by one-way ANOVA, followed by Tukey’s post hoc test for comparisons between the groups. A value of *P*<0.05 was considered to be statistically significant. 

## Results

***Studies in naïve rats***

*Two-way active avoidance task*

*Conditioned responses (avoidances)*

The animals treated with 5 mg/kg and 15 mg/kg BW TCP did not show significant increase in the number of conditioned responses during the training session, nor during the two retention tests on the 5^th^ and 12^th^ day, compared to the control group respective day. The rats treated with the highest dose of TCP (30 mg/kg BW), did not change significantly the number of active avoidances during the learning session and the short-term memory test but increased their number during the long-term memory retention test (*P*<0.05). The animals treated with TCP at a dose of 30 mg/kg BW significantly increased the number of CR when compared with groups that received TCP at doses of 5 and 15 mg/kg BW on the 12^th^ day (Figure 1). 

*Unconditioned responses (escapes)*

The group treated with the lowest dose of TCP, had significantly increased number of unconditioned responses on the 2^nd^ (*P*<0.001) and 4^th^ (*P*<0.05) day of the learning session as well as on the 5^th^ and 12^th^ days during the memory retention tests (*P*<0.05), compared to the respective day of the control group. The animals that received TCP at 15 mg/kg BW dose had increased number of UCR on the 2^nd^ training day (*P*<0.05) but had no significantly changed number of passive escapes during the memory sessions. The rats treated with the highest dose of TCP (30 mg/kg BW) had significantly increased number of UCR on the 12^th^ day during the long-term memory test (*P*<0.01), compared with saline. The group treated with TCP at dose of 30 mg/kg BW had significantly decreased number of UCR compared to TCP 5 mg/kg on the 2^nd^ learning day (*P*<0.05) and increased their number compared to TCP 15 mg/kg BW during the long-term memory test (*P*<0.05) (Figure 2). 

*T-maze*

All experimental groups treated with TCP at 5, 15, and 30 mg/kg BW significantly increased the working memory index when compared to saline (Table 1).

*Locomotor activity*

The three groups treated with TCP had significantly increased number of relative units on horizontal (*P*<0.001) and vertical (*P*<0.05) movements, compared to the control group (Figure 3).

***Studies in haloperidol challenged-rats***

*Two-way active avoidance task*

*Conditioned responses (avoidances)*

The animals treated with HP (1 mg/kg BW) and the three experimental groups with HP and TCP at 5, 15, and 30 mg/kg BW had significantly decreased number of active avoidances during the learning session as well as during the two memory retention tests on the 5^th^ and 12^th^ days compared to the saline group for the respective day. None of the three experimental groups showed significant increase in the number of conditioned responses when compared to the haloperidol control group. There is no statistically significant difference between the tolcapone treated groups (Figure 4).

*Unconditioned responses (escapes)*

The rats treated with 1 mg/kg BW HP had significantly decreased number of escapes throughout the whole training session (*P*<0.0001) and the two memory retention tests (*P*<0.0001) in comparison with the saline group for the respective day. The three experimental groups treated with HP and TCP at doses of 5, 15, and 30 mg/kg BW had significantly decreased unconditioned responses during the learning days and both memory tests in comparison with the saline group respective day. When compared with the haloperidol control group, the animals treated with HP and TCP at doses of 15 and 30 mg/kg BW had significantly increased number of escapes on the 1^st^ day of learning (*P*<0.05). There is no statistically significant difference between the tolcapone treated groups (Figure 5).

*T-maze*

The three experimental groups with haloperidol and tolcapone did not show significant increase in the working memory index when compared to both control groups (Table 2).

*Locomotor activity in haloperidol-challenged rats*

The rats given haloperidol (1 mg/kg BW) showed significant decrease in the number of horizontal movements (*P*<0.05) and non-significant decrease in vertical movements when compared to the saline group. The animals treated with HP and all doses of TCP had significantly decreased movements in the horizontal plane (*P*<0.05) when compared with the saline group but not in the vertical one (Figure 6). 

## Discussion

The major finding of this study is that in naïve rats the COMT inhibitor tolcapone at 30 mg/kg BW improves long-term memory in TWAA and spatial working memory in the T-maze task. Since the brain areas involved in these tasks are different, our results show that tolcapone improves memory by acting at different brain areas. 

The hippocampus is involved in memory processing. It is suggested that the dorsal hippocampus is associated with cognitive functions, while the ventral hippocampus is responsible for other functions like emotions, anxiety, and reward (19). In the present study, we tested the effect of COMT inhibitor tolcapone on learning and memory consolidation in the TWAA test in naïve rats and haloperidol-challenged rats. This memory task is dependent on the integrity of the hippocampus and especially its dorsal part (20). Our results suggest that this brain region might have been responsible for the mechanism by which tolcapone improves cognition. Dopamine is one of the most important mediators in the hippocampus. The dopaminergic system has a key role in the regulation of normal hippocampal function. Long term potentiation (LTP) is one possible mechanism by which hippocampal dopamine release regulates learning and memory. The dopamine receptors are cloned and characterized in five classes. They are subdivided into D1-like (D1 and D5) and D2-like (D2, D3, and D4) receptors (21). Both D1 and D2 receptors are expressed in the dorsal hippocampus (22). Dopamine usually modulates the long-lasting changes of synaptic transmission by acting on D1-like receptors. They enhance both early and late LTP in the CA1 area (23). The D2 receptors play an essential role in the regulation of hippocampal-dependent learning and memory by modulating the long-term depression in the temporal hippocampus (24). A study showed that under normal conditions the LTP is higher in the dorsal hippocampus than in the ventral hippocampus (8). It is known that tolcapone inhibits COMT activity and increases dopamine levels. So, we can speculate that the obtained results reveal the role of the hippocampal dopamine in tolcapone-induced improvement of spatial memory. Our results are consistent with previous preclinical studies using hippocampal-dependent tasks (25). 

The COMT enzyme is widely distributed in the mammalian brain but plays a more important role in the metabolism of dopamine in the rat prefrontal cortex (15) than in subcortical structures. Findings showed under basal conditions that one half of the prefrontal dopamine clearance was due to the contribution of COMT (11). This poses the questions of whether the COMT enzyme is expressed in the hippocampus and whether tolcapone may significantly increase the levels of dopamine in this brain structure. Matsumoto *et. al.* with *in situ* hybridization techniques demonstrated expression of COMT mRNA in the hippocampal dentate gyrus and the CA region of rats (26). Earlier biochemical studies also showed high COMT enzyme activity in the rat hippocampus (27). These results indirectly support our findings for a hippocampal-dependent mechanism by which tolcapone improves cognition.

Tolcapone passes the blood-brain barrier and inhibits the brain COMT activity *in vivo *(28). In our study, the dose of 30 mg/kg BW significantly increased the number of conditioned responses in long-term memory retention tests. Acquas *et. al.* have shown that this dose significantly inhibits the COMT activity (29). One limitation of our study is that tolcapone is not applied topically in the hippocampus but systematically and this might inhibit the enzyme activity in the entire brain. Based on our results we cannot be certain that tolcapone improves memory by a hippocampal-dependent mechanism, but we can suggest this mechanism is possible. This hypothesis is supported by findings of Laatikainen and coworkers who showed that tolcapone modulates dopamine metabolism in the dorsal hippocampus. The same authors showed that tolcapone improves memory in two different ways than our hippocampus-dependent memory tests – delayed rewarded alternation and spatial novelty preference tasks (16).

Noradrenaline is also involved in hippocampal memory consolidation and retrieval (30). This mediator is present in larger quantities in the hippocampus than dopamine. Laatikainen *et. al.* found that noradrenaline/dopamine ratio in this brain structure is about 24.9 in naïve rats and 21 in the presence of tolcapone (16). In order to study the role of dopamine and dopamine receptors in the mechanism of tolcapone-induced improvement of memory retention, we conducted a second series of experiments. In the haloperidol challenged rats, tolcapone treatment did not significantly increase neither the number of conditioned, nor the number of unconditioned responses when compared with the saline group. Haloperidol is a dopamine receptor antagonist that has higher affinity for the dopamine D2 receptors than D3 receptors (D2/D3 Ki ratio, 0,195) (31). The ability of haloperidol to antagonize the effect of tolcapone indicates the role of dopaminergic mediation and the D2 receptors in the observed effect. It can be speculated that D2 receptors modulated long-term depression in the dorsal hippocampus is the mechanism by which tolcapone improves memory consolidation. 

The monoaminergic neurotransmission has been identified to be important for the modulation of spatial working memory. Both noradrenaline and dopamine have an important and complementary role (32). The brain structure involved in this process is the medial prefrontal cortex (mPFC) (33). The choice behavior in the T-maze task is mediated by neuronal encoding in the mPFC (34). In our study, tolcapone in all tested doses improves spatial working memory in this task. These results indicate the role of mPFC in the mechanism by which tolcapone improves cognitive functions. Our findings are in conformity with previous studies for the involvement of mPFC in tolcapone induced improvement of memory performance. Clinical studies showed that tolcapone improves memory processed by the prefrontal cortex not only in patients with PD (13) but also in healthy humans (14). Preclinical studies using different mPFC dependent tasks also showed that COMT inhibition by tolcapone improves memory functions (15, 35). In novel object recognition task, researchers showed that tolcapone ameliorates recognition memory deficits in normal, phencyclidine-treated rats and in transgenic mice expressing the COMT-Val form, which is related with an increase in the function of the COMT enzyme (36). These results can be easily explained by the fact that in the cortex expression of the dopamine transporter is very low and the dopamine inactivation depends preferentially on COMT (37). Thus, the leading role of the COMT enzyme in inactivating cortical dopamine is supported by the findings that dopamine tissue levels are higher in the frontal cortex of COMT knockout mice (38), and COMT mRNA is highly expressed in the prefrontal cortex of human and rat brain (26). 

COMT enzyme metabolizes not only dopamine but also other catecholamines, e.g., noradrenaline. In order to distinguish which mediator is involved in tolcapone-induced improvement of spatial working memory, the rats were challenged with D2 receptor antagonist haloperidol. Our results showed that the effect of the COMT inhibitor in ameliorating memory was lost in rats with blockage of the dopamine receptors. This indicates the role of D2 receptors in the observed effect. Although D1 receptors are more abundant in the prefrontal cortex and more important for spatial working memory (39) some studies showed that D2 receptors may also be involved (40). A study found that haloperidol impairs spatial working memory in healthy humans (41).

## Conclusion

Our results demonstrate that in naïve rats the brain-penetrating COMT inhibitor tolcapone improves memory in the hippocampal-dependant TWAA task and spatial working memory in T-maze task which involves mPFC. Furthermore, our study also showed that improvement of memory functions depends on D2-like receptors since the observed memory amelioration was lost in the presence of D2/3 receptor antagonist haloperidol. 
